# Submucosal Tunneling Endoscopic Resection for Upper Gastrointestinal Subepithelial Lesions: Development of a Digital Nomogram

**DOI:** 10.1055/a-2885-6152

**Published:** 2026-06-12

**Authors:** Fatih Aslan, Serhat Ozer, Ozgur Aktas, Sevde Elif Odemis

**Affiliations:** 1 Department of Gastroenterology 52979 Koc UniversitesiIstanbulİstanbulTurkey; 2 Faculty of Medicine 52979 Koc UniversitesiIstanbulİstanbulTurkey

**Keywords:** STER, subepithelial lesion, GIST, neuroendocrine tumor, subepithelial tumors

## Abstract

**Background and Aims** Submucosal tunneling endoscopic resection (STER) is a minimally invasive technique for treating subepithelial lesions (SELs) originating from the muscularis propria. We aimed to evaluate the efficacy and safety of STER, independent predictors of procedural outcomes, and establish evidence-based patient selection criteria.

**Methodology** A retrospective analysis was conducted on patients who underwent STER for esophageal and gastric SELs between January 2017 and December 2024. En bloc resection was defined as complete dissection of the lesion as a single piece from its site of origin within the submucosal tunnel. Piecemeal retrieval referred to fragmentation of an en bloc-dissected specimen during transluminal extraction owing to size constraints. Primary outcomes included technical success, en bloc resection rates, and complication rates. Secondary outcomes assessed factors predictive of piecemeal retrieval, procedure difficulty, and complication risk. Multivariable logistic regression and receiver-operating characteristic (ROC) analysis were performed.

**Results** Among 150 patients, en bloc dissection within the submucosal tunnel was achieved in 100% of cases; en bloc retrieval through the lumen was achieved in 128 patients (85.33%), while 22 (14.67%) required piecemeal retrieval. Longitudinal length was the only independent predictor of piecemeal retrieval (aOR: 1.48 per 10 mm, 95% CI: 1.04–2.11, *p* = 0.031) and procedural difficulty (aOR = 1.07 per mm, 95% CI: 1.02–1.12, *p* = 0.003). Deep muscularis propria origin was the independent predictor of complication (aOR = 114.45, 95% CI: 30.13–434.71, *p* < 0.001). ROC analysis established optimal cut-offs: 43 mm for piecemeal retrieval (area under the curve [AUC] = 0.986), 38 mm for difficult procedures (AUC = 0.770), and 34 mm for complications (AUC = 0.739).

**Conclusions** STER demonstrates high efficacy and safety for treating muscularis propria-originating SELs. Longitudinal length is the strongest predictor of piecemeal retrieval and procedural difficulty, while a deep-layer origin predicts complications. An internally validated, freely accessible interactive risk calculator ( https://nomosmed.streamlit.app/ ) is provided as a research prototype to support preoperative risk stratification; external multicenter validation is required before routine clinical adoption.

## Introduction

Subepithelial lesions (SELs) of the upper gastrointestinal tract, including gastrointestinal stromal tumors (GISTs), leiomyomas, and neuroendocrine tumors (NETs), comprise a heterogeneous group of lesions that often pose diagnostic and therapeutic challenges. 1 2 Although most SELs are discovered incidentally during endoscopic evaluation and remain asymptomatic at diagnosis, their unpredictable biological behavior and potential for malignant transformation necessitate timely intervention. 3 4

Since its introduction in 2012, the Submucosal Tunneling Endoscopic Resection (STER), also referred to as Peroral Endoscopic Tumor Resection (POET) has been developed as a minimally invasive third-space endoscopic technique for the treatment of SELs of the esophagus and stomach. This method has become a promising therapeutic option, particularly for tumors originating from the muscularis propria (MP) layer. By creating a submucosal tunnel to access the lesion, the procedure enables en bloc resection while preserving mucosal integrity and minimizing adverse events such as perforation or infection. 1 3 5 The technique extends beyond the typical size limitations of conventional endoscopic methods, allowing safe removal of tumors up to 35 mm, and even 5–7 cm in selected cases. 2 Thus, STER/POET reflects the expanding role of third-space endoscopy in the minimally invasive, organ-sparing management of subepithelial tumors. 5 6 7 Despite the increasing use of STER for treating SELs with malignant potential such as GISTs and NETs, comprehensive data demonstrating predictors of procedural outcomes remain limited. While several studies have identified tumor size as a critical factor, the independent contribution of size versus other characteristics (tumor location, growth pattern, layer of origin) has not been clearly established through rigorous analysis.

In this context, this study aims to: (1) evaluate the efficacy and safety of the STER technique; (2) identify independent predictors of piecemeal retrieval, procedural difficulty, and complications; (3) establish evidence-based size thresholds; and (4) develop a preoperative risk stratification system to guide clinical decision-making.

## Methods

### Study Design and Setting

This retrospective cohort study was conducted at Koç University School of Medicine, Istanbul, Turkey, with institutional review board approval (Koc University 2025.241.IRB1.040), and informed consent was obtained from all patients prior to the procedure.

### Patient Selection

Consecutive patients who underwent STER for SELs originating from the MP layer between January 2017 and December 2024 were included. Inclusion criteria comprised: (1) SELs located in the esophagus or stomach (cardia, corpus, lesser curvature, antrum, and greater curvature); (2) tumors originating from the MP layer and submucosal layer confirmed by endoscopic ultrasound (EUS); (3) tumor size suitable for endoscopic resection; and (4) complete clinical and follow-up data available.

**Fig. 1:**
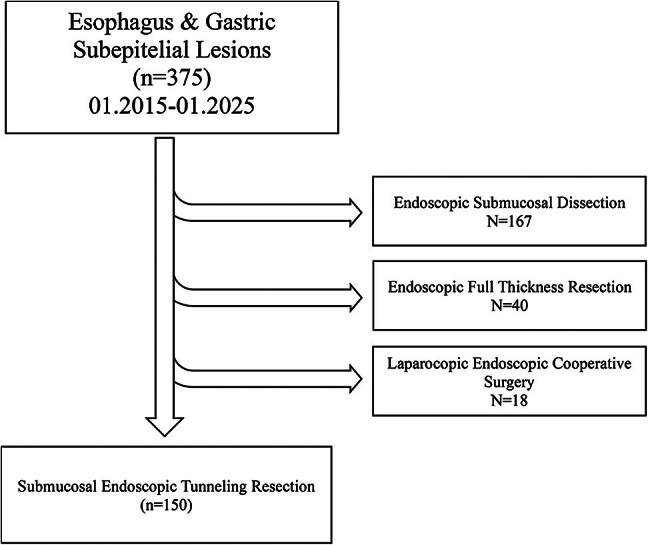
Flow chart displaying patient selection and treatment distribution.

Exclusion criteria included: (1) tumors with mucosal involvement, (2) evidence of distant metastasis, (3) significant comorbidities precluding endoscopic intervention, and (4) incomplete procedural data. Patient selection is presented in Fig. 1 .

### Preoperative Evaluation

All patients underwent contrast-enhanced computed tomography of the lesion site and EUS immediately before the procedure. The relationship of the lesions with adjacent organs and their layer of origin were documented. Lesion longitudinal and transverse dimensions were assessed by EUS using the maximal length and width measured along the long and short axes of the hypoechoic mass on the optimal scanning plane. Radial EUS was used in most cases; we acknowledge that the radial format is optimized for cross-sectional imaging and that maximal longitudinal length on radial EUS may be subject to greater measurement variability than linear EUS, particularly for elongated or irregularly shaped lesions. To mitigate this, longitudinal length was cross-checked against intraoperative endoscopic measurement and post-resection specimen measurement, with the largest of the concordant values recorded for analysis.

### STER Procedure

All procedures were performed under general anesthesia with endotracheal intubation. The standardized STER technique consisted of the following: (1) Submucosal injection: A normal saline with indigo carmine was injected 5 cm proximal to the lesion using a sclerotherapy needle (Needle Master, Olympus, Tokyo, Japan) to induce mucosal elevation. (2) Mucosal incision and tunnel creation: A linear mucosal incision was induced using either a Dual knife (Olympus KD-655L, Tokyo, Japan) or Triangle knife (Olympus KD-645L, Tokyo, Japan). A submucosal tunnel was created extending to the distal margin of the tumor. (3) Tumor dissection: After complete mobilization from the mucosa, the tumor was dissected from its origin using the same knife. (4) Retrieval: Tumors were retrieved using SNARE, ERCP basket (Olympus, Tokyo, Japan), and Roth Net retriever. (5) Tunnel irrigation and closure: The tunnel was irrigated with gentamicin-containing saline (80 mg). Closure was achieved using hemoclips (EZ Clip, Olympus, Tokyo, Japan; or Micro-Tech, Nanjing, China) or the OverStitch endoscopic suturing system (Apollo Endosurgery, Austin, TX, USA) via double-channel endoscope (Olympus GIF-2T180, Tokyo, Japan).

**Fig. 2:**
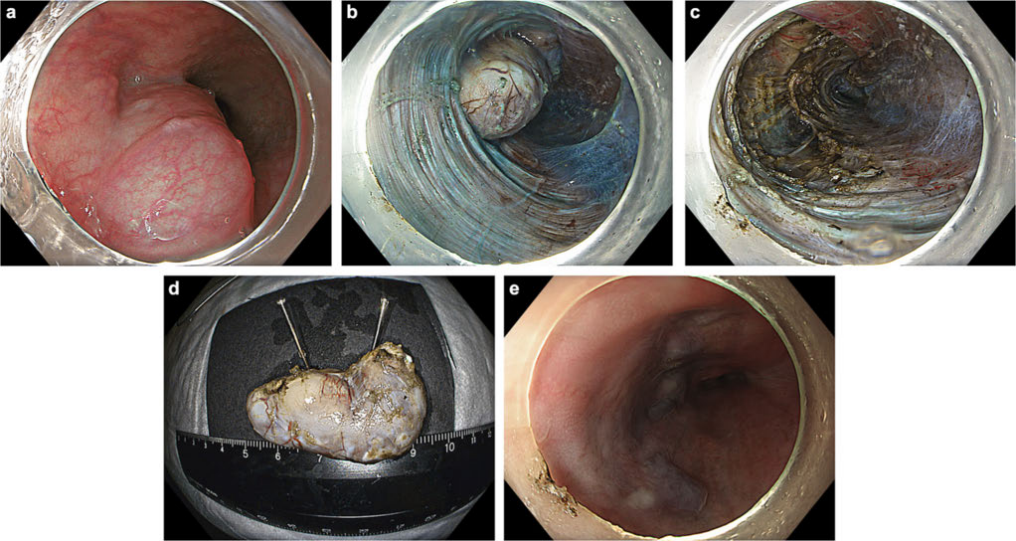
( **a** ) Endoscopic view of the subepithelial lesion located on the left lateral wall of the esophagus. ( **b** ) Intraluminal view of the lesion through the submucosal tunnel after submucosal injection and tunnel creation. ( **c** ) Postprocedural appearance of the resection site, showing the muscular layer included in the specimen and exposure of the mediastinal space. ( **d** ) Postresection view of the endoscopic view of the mucosal defect at the site where the lesion was removed. ( **e** ) Gross appearance of the resected specimen, including the muscular layer.

**Fig. 3:**
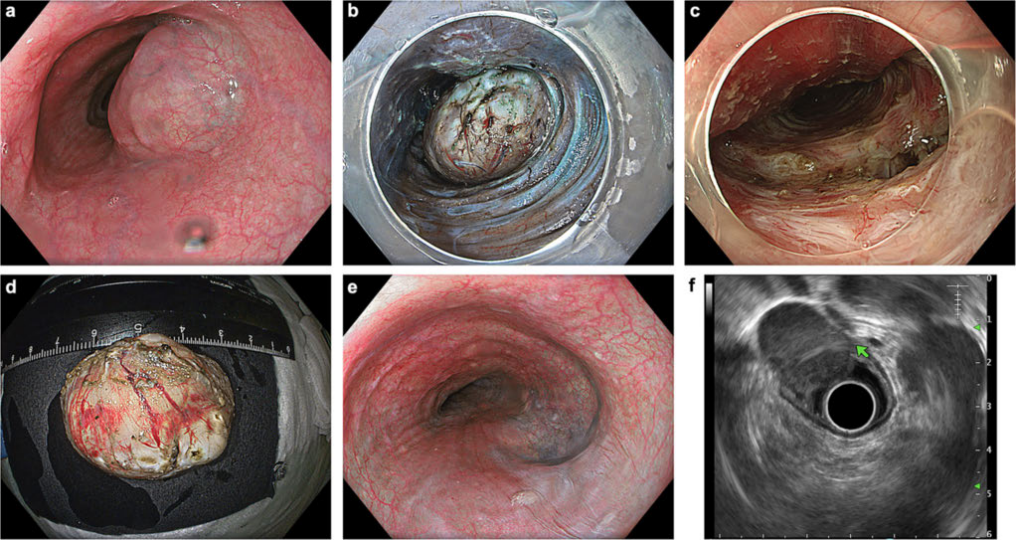
( **a** ) Endoscopic view of a subepithelial lesion located on the right lateral wall of the esophagus. ( **b** ) Intraluminal view of the lesion through the submucosal tunnel. ( **c** ) Postprocedural appearance of the resection site; since the lesion originated superficial muscular layer, the entire muscularis was not removed, and no opening into the mediastinum occurred. ( **d** ) Postresection endoscopic view of the mucosal defect at the site of the removed lesion. ( **e** ) Gross appearance of the resected specimen. ( **f** ) Radial endoscopic ultrasound image showing the origin of the lesion located on the right lateral wall of the esophagus.

To ensure consistency in data collection and presentation of our analysis, specific definitions were employed. En bloc dissection within the tunnel was defined as complete removal of the lesion as a single, intact piece from its site of origin within the submucosal tunnel. Piecemeal retrieval was defined as the need to fragment a successfully en bloc-dissected specimen during transluminal extraction through the esophagus owing to luminal size constraints. We use the term “en bloc retrieval” only for specimens that were both dissected and extracted intact through the lumen. Procedure time was calculated from the initial mucosal incision to complete tumor removal, encompassing tunnel creation, tumor dissection, and specimen retrieval. The standardized STER technique is illustrated in Fig. 2a–e , demonstrating procedural steps for a lesion originating from the deep muscle layer of the esophagus. Fig. 3a–f illustrates STER for a lesion originating from the superficial muscle layer, demonstrating the technique’s adaptability based on the tumor’s depth of origin.

### Management of Intraoperative and Postoperative Complications

Gas-related complications (including subcutaneous emphysema, pneumothorax, pneumoperitoneum) were monitored intraoperatively and postoperatively. Clinically significant pneumoperitoneum was managed by 18G needle decompression. Postoperative complications such as pleural effusion, peritonitis, and hospitalization duration were recorded.

### Outcome Measures

Primary outcomes included technical success (defined as complete tumor resection), en bloc resection rate, and procedure-related complications. Secondary outcomes comprised predictors of piecemeal retrieval, difficult procedure risk (defined as hospital stay ≥ 4 days or procedure time >60 min), and complication risk.

### Statistical Analysis

**S** tatistical analyses were performed using Stata/SE version 19.0 (StataCorp LLC, College Station, TX, USA). Normally distributed continuous variables were expressed as mean ± standard deviation (SD), whereas non-normally distributed variables were reported as median with interquartile range (IQR). Categorical variables were presented as frequencies and percentages. Comparative analyses used chi-square test or Fisher’s exact test, Mann–Whitney *U* test or Kruskal–Wallis test as appropriate. Univariable and multivariable logistic regression analyses identified the secondary outcomes. Variables with *p* < 0.10 in univariable analysis were included in the multivariable model to capture critical predictors while avoiding overfitting. Results were reported as odds ratios (ORs) with corresponding 95% confidence intervals (CIs) and *p* -values. Receiver-operating characteristic (ROC) curves and Youden’s J statistics were used to determine optimal cut-offs. Internal validation (IV) was performed using bootstrap validation ( *n* = 1000) and the Hosmer–Lemeshow (H–L) goodness-of-fit test were utilized.

## Results

### Patient and Tumor Characteristics

A total of 150 patients underwent STER during the study period ( Table 1 ), including 80 males (53.33%) and 70 females (46.67%), with a mean age of 56.26 ± 12.9 years. Tumors were located in the esophagus in 76 cases (50.67%) and in the stomach in 74 cases (49.33%).

**Table 1:** Baseline demographic, clinical, and procedural characteristics of patients undergoing submucosal tunneling endoscopic resection (STER) for upper GI subepithelial tumors.

Characteristics	Results ( *n* = 150)
Age (mean) ± SD	56.26 (12.9)
** Gender, *n* (%) **	
Male	80 (53.33)
Female	70 (46.67)
** Locations, *n* (%) **	
Esophagus	76 (50.67)
Gastric Body/Greater Curve	39 (26.0)
Lesser Curve/Cardia	35 (23.3)
** Symptoms, *n* (%) **	
Anemia	6 (4)
Dysphagia	42 (28)
Dyspepsia	87 (58)
GERD	15 (10)
** Origin, *n* (%) **	
Submucosa	3 (2.01)
Deep Muscle	63 (42.28)
Superficial Muscle	83 (55.7)
** Growth Pattern, *n* (%) **	
Presence of Extraluminal Component	14 (9.33)
No Extraluminal Component	136 (90.67)
** Pathology, *n* (%) **	
Leiomyoma	110 (73.33)
GIST	22 (14.67)
Ectopic Pancreas	11 (7.33)
Lipoma	3 (2)
Granular Cell Tumor	2 (1.33)
Glomus	1 (0.67)
Schwannoma	1 (0.67)
** Retrieval Method, *n* (%) **	
En Bloc	128 (85.33)
Piecemeal	22 (14.67)
** Closure Type, *n* (%) **	
OverStitch	17 (11.33)
Hemoclip	133 (88.67)
** Knife, *n* (%) **	
Dual	39 (26)
Triangle	111 (74)
** Postoperative Complications, *n* (%) **	
No Postoperative Complications	82 (54.67)
Emphysema	22 (14.67)
Pleural Effusion	7 (4.67)
Pneumoperitoneum	45 (30)
** Endoscopic Complications, *n* (%) **	
No Postoperative Complications	145 (96.67)
Mucosal Laceration	5 (3.33)
** Endoscopic Complications Interventions, *n* (%) **	
Hemoclip	4 (2.67)
Paracentesis	25 (16.67)
Stent	1 (0.67)
No Intervention	120 (80)
**Procedure Time (min), median (IQR)**	40 (25-60)
**Hospital stay (days), median (IQR)**	3 (2-3)
**Tumor Longitudinal Length (mm), median (IQR)**	28 (22-40)
**Tumor Transverse Length (mm), median (IQR)**	20 (15-25)

*SD: standard deviation; IQR: interquartile range; STER: submucosal tunneling endoscopic resection; GIST: gastrointestinal stromal tumor; GERD: gastroesophageal reflux disease*

Indications for endoscopy were dyspepsia (58%), dysphagia (28%), gastroesophageal reflux disease (10%), and anemia (4%). Among patients with esophageal lesions, 57% presented with dysphagia, while 2.7% of those with gastric lesions had dysphagia symptoms. All patients investigated for anemia had gastric lesions. Tumor origin within the MP was superficial in 83 cases (55.7%), deep in 63 (42.28%), and submucosal in 3 (2.01%).

The pathological distribution comprised leiomyomas (110 cases, 73.33%); GISTs (22 cases, 14.67%); ectopic pancreas (11 cases, 7.33%); lipomas (three cases, 2%); granular cell tumors (two cases, 1.33%); glomus tumors (one case, 0.67%); and schwannomas (one case, 0.67%). The median tumor longitudinal and transverse length were 28 mm (IQR: 22–40) and 20 mm (IQR: 15–25), respectively.

### Procedural Outcomes

Technical success was achieved in all cases (100%). All tumors were successfully dissected en bloc within the tunnel; en bloc retrieval through the tunnel was accomplished in 128 patients (85.33%), while piecemeal retrieval was necessary in 22 patients (14.67%). Median procedure duration was 40 min (IQR: 25–60), and median hospitalization was 3 days (IQR: 2–3).

The triangle knife was used in 111 cases (74%) and the dual knife in 39 (26%). For tunnel closure, hemoclips were used in 133 cases (88.67%) and OverStitch in 17 cases (11.33%).

### Complications Profile

Postoperative complications occurred in 68 patients (45.33%), with pneumoperitoneum being the most common (45 cases, 30%), followed by emphysema (22 cases, 14.67%) and pleural effusion (seven cases, 4.67%). Endoscopic complications were rare, occurring in only five patients (3.33%), limited to mucosal laceration.

Interventions included paracentesis in 25 cases (16.67%), hemoclip application in four (2.67%), and stent placement in 1 (0.67%). No intervention was required in 120 cases (80%).

### Comparative Analysis by Resection Type

Patients requiring piecemeal retrieval more frequently presented with dysphagia (68.2% vs. 21.1%, *p* < 0.001) and had tumors originating from the deep muscle layer (72.7% vs. 36.7%, *p* = 0.007) ( Table 2 ). Tumor size classification showed dramatic differences, with all tumors <30 mm achieving complete resection, while 89.5% of tumors ≥50 mm required piecemeal retrieval ( *p* < 0.001). Procedure duration was significantly longer for piecemeal retrieval (median 73 vs. 36 min, *p* < 0.001).

**Table 2:** Comparison of clinical, procedural, and pathological characteristics between patients undergoing complete (en bloc) versus piecemeal retrieval.

Characteristics	Complete resection ( *n* = 128)	Piecemeal retrieval ( *n* = 22)	*p* -Value*
**Age (mean) ± SD**	56.6 (13.4)	54.3 (9.1)	0.225
** Gender, *n* (%) **			0.734
Male	69 (53.9)	11 (50)	
Female	59 (46.1)	11 (50)	
** Location of STER, *n* (%) **			0.075
Gastric STER	67 (52.3)	7 (31.8)	
Esophageal STER	61 (47.7)	15 (68.2)	
** Symptoms, *n* (%) **			**<0.001**
Anemia	4 (3.1)	2 (9.1)	
Dysphagia	27 (21.1)	15 (68.2)	
Dyspepsia	85 (66.4)	2 (9.1)	
GERD	12 (9.4)	3 (13.6)	
** Origin, *n* (%) **			**0.007**
Submucosa	3 (2.3)	0	
Deep Muscle	47 (36.7)	16 (72.7)	
Superficial Muscle	77 (60.2)	6 (27.3)	
** Specific Esophageal Location, *n* (%) **			0.216
Proximal Esophagus	6 (4.7)	3 (13.6)	
Mid-Esophagus	34 (26.6)	10 (45.5)	
Distal Esophagus	21 (16.4)	2 (9.1)	
** Specific Gastric Location, *n* (%) **			0.130
Antrum	11 (8.6)	0 (0)	
Cardia	31 (24.2)	6 (27.3)	
Corpus	25 (19.5)	1 (4.5)	
** Pathology, *n* (%) **			0.169
Leiomyoma	88 (68.8)	22 (100)	
GIST	22 (17.2)	0	
Ectopic Pancreas	11 (8.6)	0	
Lipoma	3 (2.3)	0	
Granular Cell Tumor	2 (1.6)	0	
Glomus	1 (0.8)	0	
Schwannoma	1 (0.8)	0	
** Tumor Classification, *n* (%) **			**<0.001**
<30 mm	79 (61.7)	0 (0)	
30–50 mm	47 (36.7)	5 (22.7)	
>50 mm	2 (1.6)	17 (77.3)	
**Hospital Stay (days), median (IQR)**	3 (1)	3 (2)	
**Procedure Length (min), median (IQR)**	36 (30.5)	73 (42)	**<0.001**

*Chi-squared or Fisher’s exact test for categorical variables; Mann–Whitney U test for continuous variables.

### Tumor Size Stratification Analysis

Table 3 presents a stratified analysis by tumor size categories (<30, 30–50, ≥50 mm). Larger tumors were associated with increased dysphagia symptoms ( *p* < 0.001), deeper muscle origin ( *p* = 0.001), longer procedure times ( *p* < 0.001), higher complication rates ( *p* < 0.001), and prolonged hospitalization ( *p* = 0.0013). All tumors <30 mm achieved complete resection, while piecemeal retrieval was required in 9.6% of 30–50 mm tumors and 89.5% of ≥50 mm tumors.

**Table 3:** Stratified analysis of patients by tumor size categories (<30, 30–50, ≥50 mm). Differences in demographics, tumor characteristics, complications, and procedural outcomes.

Characteristics	<30 mm ( *n* = 79)	30–50 mm ( *n* = 52)	≥50 mm ( *n* = 19)	*p* -Value*
**Age (mean) ± SD**	56.21 (13.74)	56.33 (11.92)	56.28 (12.49)	0.532
** Gender, *n* (%) **				0.854
Male	36 (45.6)	24 (46.2)	10 (52.6)	
Female	43 (54.4)	28 (53.8)	9 (47.4)	
** Location of STER, *n* (%) **				0.259
Gastric STER	44 (55.7)	22 (42.3)	8 (42.1)	
Esophageal STER	35 (44.3)	30 (57.7)	11 (57.9)	
** Symptoms, *n* (%) **				**<0.001**
Anemia	3 (3.8)	1 (1.9)	2 (10.5)	
Dysphagia	9 (11.4)	21 (40.4)	12 (63.2)	
Dyspepsia	60 (75.9)	24 (46.2)	3 (15.8)	
GERD	7 (8.9)	6 (11.5)	2 (10.5)	
** Origin, *n* (%) **				**<0.001**
Submucosa	1 (1.3)	2 (3.8)	0 (0)	
Deep Muscle	22 (27.8)	26 (50)	15 (78.9)	
Superficial Muscle	55 (69.6)	24 (46.2)	4 (21.1)	
** Specific Esophageal Location, *n* (%) **				0.368
Proximal Esophagus	14 (17.7)	8 (15.4)	1 (5.3)	
Mid-Esophagus	18 (22.8)	18 (34.6)	8 (42.1)	
Distal Esophagus	3 (3.8)	4 (7.7)	2 (10.5)	
** Specific Gastric Location, *n* (%) **				**0.038**
Antrum	10 (12.7)	1 (1.9)	0	
Cardia	16 (20.3)	16 (30.8)	5 (26.3)	
Corpus	18 (22.8)	5 (9.6)	3 (15.8)	
** Pathology, *n* (%) **				**0.024**
Leiomyoma	49 (62)	43 (82.7)	17 (89.5)	
GIST	15 (19)	5 (9.6)	2 (10.5)	
Ectopic Pancreas	11 (13.9)	0	0	
Others	6 (7.6)	4 (7.7)	0	
** Retrieval Method, *n* (%) **				**<0.001**
Complete (En bloc)	79 (100)	47 (90.4)	2 (10.5)	
Piecemeal	0	5 (9.6)	17 (43)	
**Procedure Time (min), median (IQR)**	30 (22)	44 (31)	72 (43)	**<0.001**
** Prolonged Procedure, *n* (%) **				**<0.001**
<65 min	72 (9.1)	41 (78.8)	7 (36.8)	
>65 min	7 (8.9)	11 (21.2)	12 (63.2)	
**Presence of Postoperative Complications**	24 (30.4)	26 (50)	18 (94.7)	**<0.001**
**Emphysema**	4 (5.1)	8 (15.4)	10 (52.6)	**<0.001**
**Pleural Effusion**	1 (1.3)	4 (7.7)	2 (10.5)	0.101
**Pneumoperitoneum**	19 (24.1)	16 (30.8)	10 (52.6)	0.050
**Endoscopic Complications**	1 (1.3)	2 (3.8)	2 (10.5)	0.126
**Endoscopic Complications Interventions**	7 (8.9)	10 (19.2)	13 (68.4)	**<0.001**
**Closure Type**	13 (16.5)	3 (5.8)	1 (5.3)	0.113
**Knife Type**	61 (77.2)	37 (71.2)	13 (68.4)	0.622
**Hospitalization Duration in Days (median) (IQR)**	2 (2)	3 (1)	3 (3)	**0.001**

*Chi-squared or Fisher’s exact test for categorical variables; Kruskal–Wallis for continuous variables.

### Univariable Analysis

In univariable analysis for piecemeal retrieval, both tumor dimensions were highly significant: longitudinal length (OR = 1.35 per 10 mm, 95% CI: 1.16–1.57, *p* < 0.001) and transverse length (OR = 1.53 per 10 mm, 95% CI: 1.29–1.81, *p* < 0.001). Additional significant predictors included extraluminal growth pattern (OR = 8.07, 95% CI: 2.49–26.18, *p* < 0.001), deep-layer origin (OR = 4.74, 95% CI: 1.73–12.98, *p* = 0.002), and esophageal location showed a trend toward significance (OR = 2.35, 95% CI: 0.90–6.16, *p* = 0.081). For difficult procedures, both longitudinal (OR = 1.08, 95% CI: 1.05–1.12, *p* < 0.001) and transverse length (OR = 1.14, 95% CI: 1.08–1.21, *p* < 0.001) were significant, along with esophageal location (OR = 2.14, *p* = 0.033), extraluminal growth (OR = 3.09, *p* = 0.048), and deep-layer origin (OR = 2.16, *p* = 0.031). For complications, longitudinal (OR = 1.08, *p* < 0.001) and transverse length (OR = 1.13, *p* < 0.001), esophageal location (OR = 2.04, *p* = 0.033), and deep-layer origin (OR = 108.29, 95% CI: 32.31–362.89, *p* < 0.001) were significant, while growth pattern was not ( *p* = 0.878).

### Multivariable Analysis

For piecemeal retrieval, after adjusting for both dimensions, location, growth pattern, and layer depth, longitudinal tumor length remained independently significant (adjusted OR [aOR] = 1.48, 95% CI: 1.04–2.11, *p* = 0.031), while transverse length showed a trend toward significance (aOR = 1.55, 95% CI: 0.97–2.47, *p* = 0.067). Notably, the strong univariable associations with growth pattern (OR = 8.07), layer depth (OR = 4.74), and location (OR = 2.35) were no longer significant after adjustment ( *p* = 0.691, *p* = 0.658, and *p* = 0.268, respectively), indicating these associations were confounded by tumor dimensions; larger tumors tend to be extraluminal and originate from deeper layers. For difficult procedures, longitudinal length was the only dimension that remained independently significant (aOR = 1.07, 95% CI: 1.02–1.12, *p* = 0.003), while transverse length lost significance after adjustment ( *p* = 0.461). Location, growth pattern, and layer depth were also not significant in the multivariable model ( *p* = 0.143, *p* = 0.943, and *p* = 0.932, respectively). For complications, deep-layer origin remained the dominant independent predictor (aOR = 114.45, 95% CI: 30.13–434.71, *p* < 0.001), while both longitudinal length (aOR = 1.05, *p* = 0.091) and transverse length (aOR = 1.00, *p* = 0.939) lost significance after adjustment. This indicates that layer depth has a specific, independent association with complications that is not confounded by tumor dimensions; deep MP origin substantially increases complication risk regardless of tumor size. The results of univariable and multivariable logistic regression are displayed in Table 4 .

**Table 4:** Univariable and multivariable regression analysis for procedural outcomes.

Outcome/predictor	Univariable OR (95% CI)	*p* -Value	Adjusted multivariable OR (95% CI)	*p* -Value
**Piecemeal Retrieval** ^+^				
Longitudinal (per 10 mm)	1.35 (1.16–1.57)	**<0.001**	1.48 (1.04–2.11)	**0.031**
Transverse (per 10 mm)	1.53 (1.29–1.81)	**<0.001**	1.55 (0.97–2.47)	0.067
Location (Esophagus)	2.35 (0.90–6.16)	0.081	8.70 (0.19–401.3)	0.268
Growth (Extraluminal)	8.07 (2.49–26.18)	**<0.001**	2.27 (0.04–129.8)	0.691
Layer (Deep MP)	4.74 (1.73–12.98)	**0.002**	2.36 (0.05–104.7)	0.658
Difficult Procedure* ^+^				
Longitudinal (per mm)	1.08 (1.05–1.12)	**<0.001**	1.07 (1.02–1.12)	**0.003**
Transverse (per mm)	1.14 (1.08–1.21)	**<0.001**	1.03 (0.95–1.12)	0.461
Location (Esophagus)	2.14 (1.06–4.32)	**0.033**	1.83 (0.82–4.11)	0.143
Growth (Extraluminal)	3.09 (1.01–9.47)	**0.048**	0.95 (0.23–3.95)	0.943
Layer (Deep MP)	2.16 (1.07–4.36)	**0.031**	0.96 (0.39–2.38)	0.932
**Complications** ^++^				
Longitudinal (per mm)	1.08 (1.05–1.12)	**<0.001**	1.05 (0.99–1.11)	0.091
Transverse (per mm)	1.13 (1.07–1.19)	**<0.001**	1.00 (0.89–1.13)	0.939
Location (Esophagus)	2.04 (1.06–3.92)	**0.033**	2.48 (0.69–8.90)	0.164
Layer (Deep MP)	108.29 (32.31–362.89)	**<0.001**	114.45 (30.13–434.71)	**<0.001**

*OR: odds ratio; CI: confidence interval; MP: muscularis propria. Bold P-values indicate statistical significance (P < 0.05).*

*Difficult procedure defined as hospital stay ≥4 days OR procedure time >60 min.

*+Adjusted for: Longitudinal Length, Transverse Length, Location, Growth Pattern, Layer Depth.*

*^++^ Adjusted for: Longitudinal Length, Transverse Length, Location, Layer Depth (Growth Pattern not significant in univariable analysis, P* = *0.878).*

### ROC Analysis and Optimal Cut-offs

**Fig. 4:**
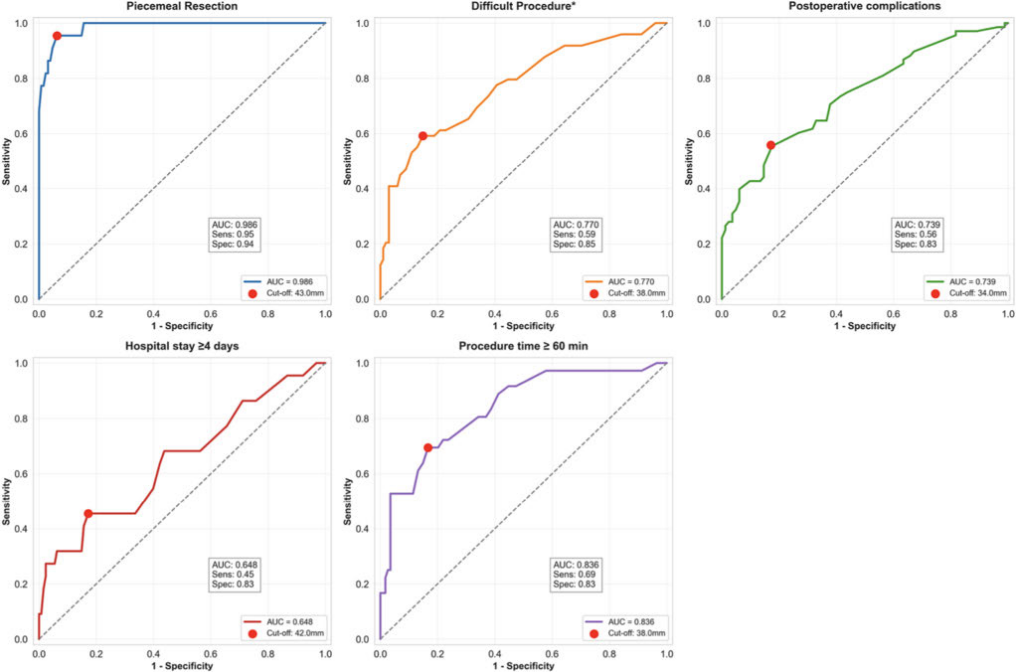
Receiver-operating characteristic (ROC) curves for longitudinal tumor length predicting piecemeal retrieval, complication risks, STER difficulty, procedure duration ≥60 min and hospital stay ≥4 days.

Table 5 presents the optimal longitudinal tumor length cut-off values for predicting various adverse outcomes. The most clinically relevant finding was the cut-off of 43 mm for predicting piecemeal retrieval, which demonstrated excellent discriminatory capability with an area under the curve (AUC) of 0.986, a sensitivity of 95.5%, and a specificity of 93.8%. IV by 1,000-iteration bootstrap confirmed an apparent AUC of 0.986 with minimal optimism, and the H–L test yielded a p value of 0.66, consistent with adequate calibration. Additional cut-offs included 38 mm for difficult procedures: >60 min or hospital stay ≥ 4 days (AUC = 0.770, sensitivity 59.2%, specificity 85.1%, H–L test *p* = 0.83), 34 mm for postoperative complications (AUC = 0.739, sensitivity 55.9%, specificity 82.9%, H–L test *p* = 0.9); however, given that layer depth is also an independent predictor of complication, size alone provides moderate but not complete discriminatory ability for this outcome. Fig. 4 displays the ROC curves for these different outcome measures. IV of ROC analysis was conducted using a bootstrap methodology with 1000 repetitions to assess the reliability of our findings.

**Table 5:** Optimal longitudinal tumor length cut-off values predicting adverse procedural outcomes using ROC analysis.

Outcome measure	Optimal cut-off longitudinal tumor length (mm)	AUC	Sensitivity	Specificity
Piecemeal Retrieval	43.0	**0.986**	**0.95**	**0.94**
Difficult Procedure*	38.0	**0.77**	0.59	0.85
Postoperative complications	34.0	**0.739**	0.56	0.83
Hospital stay ≥4 days	42.0	**0.648**	0.45	0.83
Procedure time ≥ 60 min	38.0	**0.836**	0.69	0.83

AUC: area under the receiver-operating characteristics curve; ROC: receiver-operating characteristics. Cut-offs determined using Youden’s J statistic. *Difficult procedure defined as hospital stay ≥4 days OR procedure time >60 min.

### Risk Stratification

**Fig. 5:**
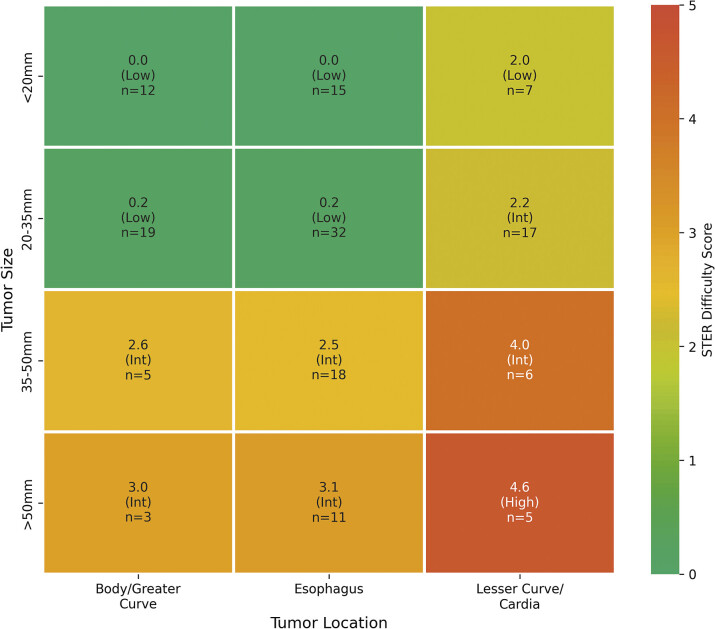
Heatmap for STER decision matrix, displaying difficulty scores stratified by tumor size and location. The heatmap color gradient (green to red) represents increasing STER Difficulty Scores from 0 to 5. Each cell shows the mean difficulty score, risk category (Low/Int/High), and sample size (n). Tumors <35 mm in favorable locations demonstrate low difficulty scores, while larger tumors (>50 mm) in high-risk locations (lesser curve/cardia) show the highest difficulty scores. Our interactive calculator displays the heatmap as per-patient parameters are adjusted which can be accessed: https://nomosmed.streamlit.app/

Based on a combination of univariable logistic regression findings and prior clinical rationale, we developed a STER Difficulty Score incorporating size >35 mm (two points), extraluminal growth pattern (three points), and high-risk location (two points). Although extraluminal growth and high-risk location did not retain independent statistical significance after adjustment for tumor dimensions, they were retained in the score on the basis of strong univariable associations, established clinical relevance for procedural planning, and the score’s purpose as a pragmatic bedside aid rather than a parsimonious statistical model. Table 6 represents the risk stratification summary. Low-risk patients (0–2 points, *n* = 126) demonstrated a piecemeal retrieval rate of 7.9% and complication rate of 38.9%. Intermediate-risk patients (3–4 points, *n* = 13) had piecemeal retrieval rate of 38.5% and complication rate of 61.5%. High-risk patients (≥5 points, *n* = 11) showed piecemeal retrieval rate of 63.6% and complication rate of 100%. A heatmap of STER Decision Matrices is displayed in Fig. 5 .

**Table 6:** Risk stratification summary by submucosal tunnel endoscopic resection (STER) difficulty score.

Risk group	N	Piecemeal rate	Complication rate	Mean longitudinal length (mm)
Low (0–2 points)	126	7.9%	38.9%	29.7
Intermediate (3–4 points)	13	38.5%	61.5%	56.7
High (≥5 points)	11	63.6%	100%	53.7

*STER Difficulty Score: Size >35 mm (+2 points), Extraluminal growth (+3 points), High-risk location (+2 points). Maximum: 7 points.*

### Interactive Risk Calculator

**Fig. 6:**
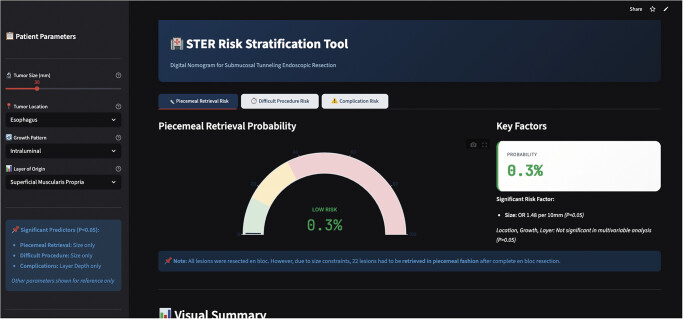
Interactive Digital Risk Stratification Tool for STER. The web-based platform (available at: ( https://nomosmed.streamlit.app/ ) translates the multivariable regression models into a user-friendly interface for real-time clinical decision support. The tool is presented as an internally validated prototype intended for research purposes; external multicenter validation is required before any clinical adoption.

For research purposes only we developed an interactive digital risk calculator incorporating the multivariable regression coefficients of three outcomes: piecemeal retrieval, difficult procedure, and complication risk. The tool is freely accessible at https://nomosmed.streamlit.app/ and allows clinicians to input patient-specific parameters (tumor size, location, growth pattern, layer depth) to obtain probability estimates for each outcome ( Fig. 6 ). This calculator is presented as an internally validated prototype intended to support hypothesis generation and discussion of preoperative planning; external multicenter validation in independent cohort is required before any clinical adoption.

## Discussion

Third-space endoscopy has redefined the therapeutic landscape for upper gastrointestinal SELs, transitioning from surgical enucleation toward organ-sparing endoscopic resection. Our study, representing one of the largest single-center experiences with STER of 150 consecutive procedures, provides a rigorous multivariable analysis that challenges traditional thresholds and introduces a framework for patient selection through an internally validated interactive nomogram prototype.

Our findings indicate that STER is safe and effective, achieving high technical success with an acceptable safety profile. In our cohort, en bloc retrieval rate of 85.33% and a technical success rate of 100% were observed, consistent with previously reported data, including the pooled en bloc resection rate of 91.5% in a meta-analysis of more than 2900 patients by Tun et al. and the 90.6% rate reported by Chen et al. 8 9 These findings support that, in experienced centers, the applicability of STER across a range of tumor sizes and locations may increase, reflecting the established volume-outcome relationship in interventional endoscopy.

A nuanced distinction needs to be made between en bloc resection (dissecting the tumor intact from the muscle) and en bloc retrieval (extracting the specimen intact through the esophageal lumen). While we achieved a 100% success in dissecting the lesions intact within the tunnel, 14.67% required piecemeal retrieval. This “en bloc paradox” is supported by Lin et al., who noted that for lesions exceeding 5.0 cm spatial constraints often necessitate segmentation for extraction, 10 referring to the anatomical limitations of the esophageal lumen and upper esophageal sphincter. 11 12 For benign leiomyomas, which constituted the majority of our cohort (73.33%), this distinction is largely academic, as piecemeal retrieval does not compromise oncological long-term outcomes and the primary goal of resection is often symptomatic relief. 11 Conversely, for GISTs (14.67%), the 43-mm threshold serves as a vital clinical consideration, as fragmentation may breach oncological principles regarding capsule integrity. 13 In our cohort, 89.5% of tumors larger than 50 mm required piecemeal retrieval, suggesting that for large suspected GISTs, a surgical approach may offer oncological assurance regarding R0 en bloc retrieval and adequate pathological assessment. 14

Longitudinal tumor length emerged as an independent predictor of both piecemeal retrieval and procedural difficulty. Previous reports indicated 35 mm constitutes a meaningful threshold for piecemeal resection; however, additional factors influencing piecemeal retrieval have also been demonstrated. 2 Our findings suggest that mechanical constraints within the cylindrical submucosal tunnel such as friction and traction forces acting on the tumor during extraction are primarily related to longitudinal tumor length rather than width, as the tubular anatomy of the upper esophagus has limited capacity to accommodate increased length. Chen et al. reported that a tumor size ≥30 mm and irregular tumor shape were independent predictors of piecemeal resection ( *p* = 0.032 and *p* = 0.029, respectively). 15 In addition, a comparative study by Tan et al., evaluating STER versus endoscopic full-thickness resection, demonstrated that tumor size was the principal determinant of treatment success irrespective of the endoscopic modality used. 3 Defining clear size thresholds may therefore support more predictable clinical decision-making.

Our ROC analysis established a longitudinal length 43 mm as the definitive threshold for predicting the necessity of piecemeal retrieval. This finding is supported by Lin et al., who identified a 4.10 cm cut-off for piecemeal retrieval, 4.35 cm for difficult procedures, and 4.75 cm for adverse events and ICU transfers. 10 Additionally, our study identified 38-mm threshold for procedural difficulty and a trend toward higher difficulty in esophageal tumors (OR: 2.35) compared to gastric locations, aligning with the limitations of the narrow esophageal lumen, which limits instrument maneuverability and distal dissection within the submucosal tunnel. 16

Regarding procedural safety, our postoperative complication rate of 45.33% was predominantly due to CO _2_ insufflation associated events such as pneumoperitoneum (30%), and emphysema. Crucially, 80% of our patients required no intervention, and only 16.67% had needle decompression. This profile is consistent with the 17.8% pooled adverse event rate in the major meta-analysis and the 18.8% rate reported by another study. 17 Our adjusted multivariable regression model identified deep MP origin as the primary determinant of safety, confirming that the anatomical plane of dissection is more critical for safety than size alone. Dissecting a tumor from the deep MP plane, originating from the outer longitudinal layer, markedly removes the muscular wall, leaving adventitia and thin serosa as a barrier, 18 supporting significance of adoption of advanced closure techniques. However, we used hemoclips in 88.67% of cases and OverStitch in 11.33%, with the latter being preferred in large tunnel entry size to provide more secure closure.

Traditional surgical approaches, including laparoscopic wedge resection and thoracoscopic enucleation (TE), have long been standard treatments. 13 Comparative studies of STER with video-assisted TE demonstrated that in terms of efficacy, both modalities were comparable for esophageal submucosal tumors; however, STER was found to be superior to video-assisted TE due to its shorter operation time, decreased cost, and safer profile. 19 Similar superior safety profiles were displayed in other studies, 13 19 20 21 22 including a retrospective study reporting shorter operating time and lower adverse event rates. 20 Although Chai et al. reported that STER is recommended for submucosal tumors <20 mm and that video-assisted TE should be preferred for tumors with a transverse diameter >35 mm, 19 our findings along with other reports 10 23 indicate that STER safely be extended to selected large lesions in experienced centers.

Prior tissue sampling represents another relevant consideration, albeit not a primary variable in our cohort, recent Western data found that 82.3% of U.S. patients had prior sampling, resulting in submucosal fibrosis in 19.6% cases. 14 The fibrosis observed was significantly associated with the need for transmural resection and longer procedural times. Our findings, combined with the Western data, suggest that if a lesion is highly suspicious of leiomyoma and the STER difficulty score is low, avoiding preresection sampling may optimize the surgical plane, reduce procedural complexity and adverse events.

The influential aspect of our research is the transition from static, consensus-based rules to a dynamic, personalized risk model. While Xiang et al. proposed a digital nomogram for giant esophageal lesions, their model primarily focused on difficulty based on age and maximal diameter. 12 Our interactive digital nomogram ( https://nomosmed.streamlid.app/ ) expands this by incorporating layer depth and anatomical location to provide separate probabilities for piecemeal retrieval, difficulty, and complications. It is worth mentioning that our interactive model also includes heatmap and STER difficulty score, allowing the user to stratify patients into actionable risk groups. We emphasize that this calculator is an internally validated prototype; pending external multicenter validation, the tool is intended for research and educational purposes only.

Several limitations merit consideration. The retrospective single-center design may limit the generalizability of our findings; longer follow-up is needed to assess oncological outcomes, particularly for GIST cases, and all procedures were conducted by a single advanced endoscopist (F.A.). The complication-prediction model is also constrained by the relative sparsity of complications among lesions originating from the superficial MP, which produces a wide CI around the deep MP OR. While the qualitative conclusion that deep MP origin is an independent predictor of complications is supported by the robust point estimate and the consistency of the directional association across analyses, the absolute magnitude of the effect should be interpreted with appropriate caution and refined in larger multicenter cohorts. The interactive nomogram has only been internally validated by bootstrap; external validation in independent multicenter cohorts is required before clinical adoption. Considering these limitations, future research should prospectively validate the size thresholds in multi-center cohorts, develop comprehensive risk prediction models, and with a larger patient populations, test machine-learning algorithms in comparative analyses against traditional tools to determine the best algorithm for patient selection.

In conclusion, STER has proven to be an effective and safe minimally invasive therapeutic option for the treatment of SELs originating from the submucosa and MP layers. Preoperative knowledge of tumor localization and dimensions may contribute significantly to appropriate patient selection and facilitate complete and en bloc resection of lesions. The integration of our internally validated state-of-the-art interactive risk calculator, pending external multicenter validation, supports tailored organ-sparing intervention for each unique patient profile.
